# Supplementary material for: Association between Nafamostat Mesylate and In-Hospital Mortality in Patients with Coronavirus Disease 2019: A Multicenter Observational Study
Source: J Clin Med. 2021 Dec 26;11(1):116. doi: 10.3390/jcm11010116 (PMC8745709; doi:10.3390/jcm11010116)
Supplement: Supplementary file 1 [file jcm-11-00116-s001.zip › jcm-1474789-supplementary.pdf]

Table S1. The International Classification of Diseases, Tenth Revision (ICD-10) codes.

Disease	ICD-10 codes
COVID-19	U071
Comorbidities at the admission	
Cancer	C00-C43, C45-C97
Chronic lung disease	J40-J47
Ischemic heart disease	I20-I25
Heart failura	I50, I110
Arrhythmia	I44, I45, I47–I49
Hypertension	I10, I11, I12, I13, I15, I674
Diabetes	E10-E14
Cerebrovascular disease	I60-I69
Peripheral artery disease	I702, I739
Chronic kidney disease	N18
End stage renal disease	N185, N19
Dementia	F00–F03, F051, G30
Cirrhosis	K703, K717, K743, K744, K745, K746
Dyslipidaemia	E78
Deficiency anemia	D50
Heart failure/cardiomyopathy	I11.0, I13.x(except I13.1), I42.x, I43.x, I50.x
Ischemic stroke/TIA/systemic thromboembolism	G45.x (except G45.4), I63.x, I69.3, I74.x
Hemorrhagic/ unspecified stroke	I60.x, I61.x, I62.x, I64.9, I69.0, I69.1, I69.2
Other vascular disease	E10.5, E11.5, E12.5, E13.5, E14.5, I65.x, I66.x, I67.x(except I67.6), I68.x, I69.4, I69.8, I70.x, I71.x, I72.x, I73.x, I75.x, I76.x, I77.x, I79.x (except I79.8)

Table S2. Summary of matched imputed datasets for in-hospital mortality (median).

	Nafamostat mesylate	Control
Number of patients	104	391
Age (year), median (IQR)	70.1 (70.0-70.2)	71.8 (71.3-72.1)
Male (%)	36 (34.6)	144 (36.8)
Charlson comorbidity index (%)		
0	48 (46.2)	176 (45.0)
1	11 (10.6)	43 (11.0)
2	24 (23.1)	95 (24.3)
3	9 (8.7)	33.5 (8.6)
≥4	12 (11.5)	43 (11.0)
Diabetes, (%)	39 (37.5)	143 (36.6)
Ischemic heart disease, (%)	6 (5.8)	24.5 (6.3)
Cirrhosis, (%)	1 (1.0)	5 (1.3)
Chronic lung disease, (%)	21 (20.1)	78 (19.9)
Cancer, (%)	12 (11.5)	46 (11.8)
Chronic kidney disease, (%)	22 (21.2)	71 (18.2)
Body mass index (%)		
< 18.5	11 (10.6)	43 (11.0)
18.5 – 25	61 (58.7)	231 (59.1)
25 – 30	21 (20.2)	75 (19.2)
≥ 30	10 (9.6)	41 (10.5)
Smoking, (%)	37 (35.6)	137 (35.0)
Japan Coma Scale, (%)		
0 (clear)	74 (71.2)	278 (71.1)
1-3 (delirium)	21 (20.2)	80 (20.5)
10-30 (somnolence)	4 (3.8)	16 (4.1)
100-300 (coma)	4 (3.8)	16 (4.1)
Warfarin, (%)	1 (1.0)	7 (1.8)
DOAC, (%)	4 (3.8)	15 (3.8)
Antiplatelets, (%)	9 (8.7)	35 (9.0)
Interhospital transfer, (%)	15 (14.4)	58 (14.8)
Hospital volume (beds, n)		
<200	3 (2.9)	12 (3.1)
200-400	36 (34.6)	148 (37.9)
≥ 400	65 (62.5)	233 (59.6)
ICU admission, (%)	31 (29.8)	106 (27.1)
Initial antibiotics, (%)	25 (24.0)	85.5 (21.9)
Heparin, (%)	13 (12.5)	45 (11.5)
Daluteparin, (%)	0	2 (0.5)
Dobutamine, (%)	0	3 (0.8)
Noradrenalin, (%)	10 (9.6)	31 (7.9)
Vasopressin, (%)	1 (1.0)	3 (0.8)
Steroids		
Dexamethasone, (%)	20 (19.2)	72 (18.4)
Other steroids, (%)	18 (17.3)	65 (16.6)
Blood transfusion (%)		
Red blood cells, (%)	2 (1.9)	7 (1.8)
Platelets, (%)	1 (1.0)	2 (0.5)
Fresh frozen plasma, (%)	0	3 (0.8)
Oxygen therapy, (%)	55 (52.9)	208 (53.2)
NPPV, (%)	1 (1.0)	5 (1.3)
Mechanical ventilation, (%)	12 (11.5)	42 (10.7)
IRRT, (%)	16 (15.4)	50 (12.8)
CRRT, (%)	2 (1.9)	5 (1.3)
ECMO, (%)	0	3 (0.8)
